# Crystal structure of 4-({(1*E*,2*E*)-3-[3-(4-fluoro­phen­yl)-1-isopropyl-1*H*-indol-2-yl]allyl­idene}amino)-5-methyl-1*H*-1,2,4-triazole-5(4*H*)-thione

**DOI:** 10.1107/S2056989015020101

**Published:** 2015-10-31

**Authors:** Ajaykumar D. Kulkarni, Md. Lutfor Rahman, Mashitah Mohd. Yusoff, Huey Chong Kwong, Ching Kheng Quah

**Affiliations:** a Department of Chemistry, KLS’s Gogte Institute of Technology, Jnana Ganga, Udyambag, Belagavi 590 008 Karnataka, India; b University Malaysia Pahang, Faculty of Industrial Sciences and Technology, 26300 Gambang, Kuantan, Pahang, Malaysia; c School of Chemical Sciences, Universiti Sains Malaysia, 11800 USM, Penang, Malaysia; d X-ray Crystallography Unit, School of Physics, Universiti Sains Malaysia, 11800 USM, Penang, Malaysia

**Keywords:** crystal structure, 1,2,4-triazole-5(4*H*)-thione, indole, Schiff base, N—H⋯S hydrogen bonds, C—H⋯π and π–π inter­actions

## Abstract

The title compound, C_23_H_22_FN_5_S, exists in a *trans* conformation with respect to the methene C=C and the acyclic N=C bonds. The 1,2,4-triazole-5(4*H*)-thione ring makes dihedral angles of 88.66 (9) and 84.51 (10)°, respectively, with the indole and benzene rings. In the crystal, mol­ecules are linked by pairs of N—H⋯S hydrogen bonds, forming inversion dimers with an *R*_2_^2^(8) ring motif. The dimers are linked *via* C—H⋯π inter­actions, forming chains along [1-10]. The chains are linked *via* π—π inter­actions involving inversion-related triazole rings [centroid–centroid distance = 3.4340 (13) Å], forming layers parallel to the *ab* plane.

## Abstract 2

The title compound exists in a *trans* conformation with respect to the methene C=C and the acyclic N=C bonds, with the 1,2,4-triazole-5(4*H*)-thione ring almost normal to the indole and benzene rings. In the crystal, mol­ecules are linked by pairs of N—H⋯S hydrogen bonds, forming inversion dimers with an 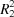
(8) ring motif.

## Chemical context   

The synthesis and functionalization of indoles has been a major area of focus for researchers for several decades. Indoles are of great importance in view of their natural occurrence as a prominent sub-structure of a large number of alkaloids (Somei & Yamada, 2003 ▸; Hibino & Choshi, 2002 ▸) and wide-ranging biological activities (Gribble, 1995 ▸). They also constitute an important moiety of various drugs. In addition, 1,2,4-triazoles are an important class of heterocyclic compounds which are well known for their potential anti­microbial properties. Substituted 1,2,4-triazoles are associated with diverse biological activities such as fungicidal, anti­microbial, anti­convulsant and anti­viral activities (Walser *et al.*, 1991 ▸; Eweiss *et al.*, 1986 ▸; Bhat *et al.*, 2001 ▸; Kitazaki *et al.*, 1996 ▸; Todoulou *et al.*, 1994 ▸). The proper design of indoles and triazoles can be used to prepare Schiff bases. The wide spectrum of biological applications of 1,2,4-triazoles prompted us to synthesize Schiff bases derived from triazole and indole derivatives. The formation of the azomethine functional group CH=N is thought to be the main reason for the biological properties of Schiff bases. We have reported a number of metal complexes of Schiff bases, recently, which possess very good anti­microbial properties (Kulkarni *et al.*, 2009*a* ▸,*b* ▸, 2011 ▸).

## Structural commentary   

**Figure 1:**
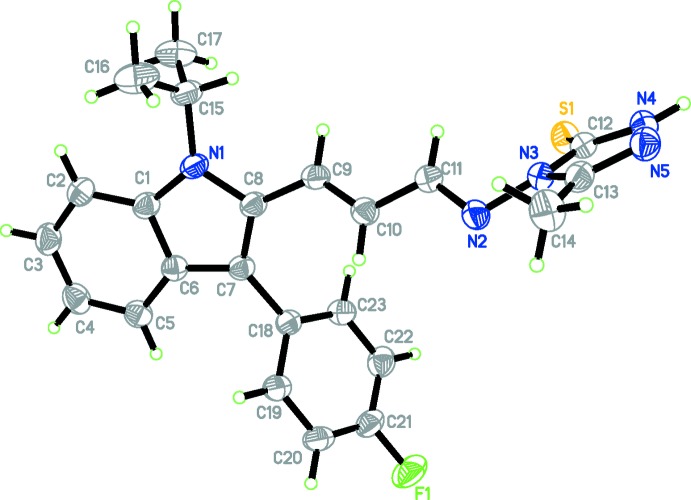
The mol­ecular structure of the title compound, with atom labelling. Displacement ellipsoids are drawn at the 30% probability level.

The title compound, Fig. 1 ▸, exists in a *trans* conformations with respect to the methene C9=C10 [1.322 (2) Å] and acyclic N2=C11 bonds [1.278 (2) Å]. The triazole ring is almost planar [maximum deviation of 0.011 (2) Å for atom C13], as is the indole ring [maximum deviation of 0.031 (2) Å for atom C4]. The triazole ring is almost normal to both the indole and benzene rings with dihedral angles of 88.66 (9) and 84.51 (10)°, respectively, while the indole and benzene ring are inclined to one another by 61.25 (8)°. The bond lengths and angles in the triazole-thione moiety of the title mol­ecule are comparable to those reported for related compounds (Fun *et al.*, 2008 ▸; Goh *et al.*, 2009 ▸; Asad *et al.*, 2010 ▸).
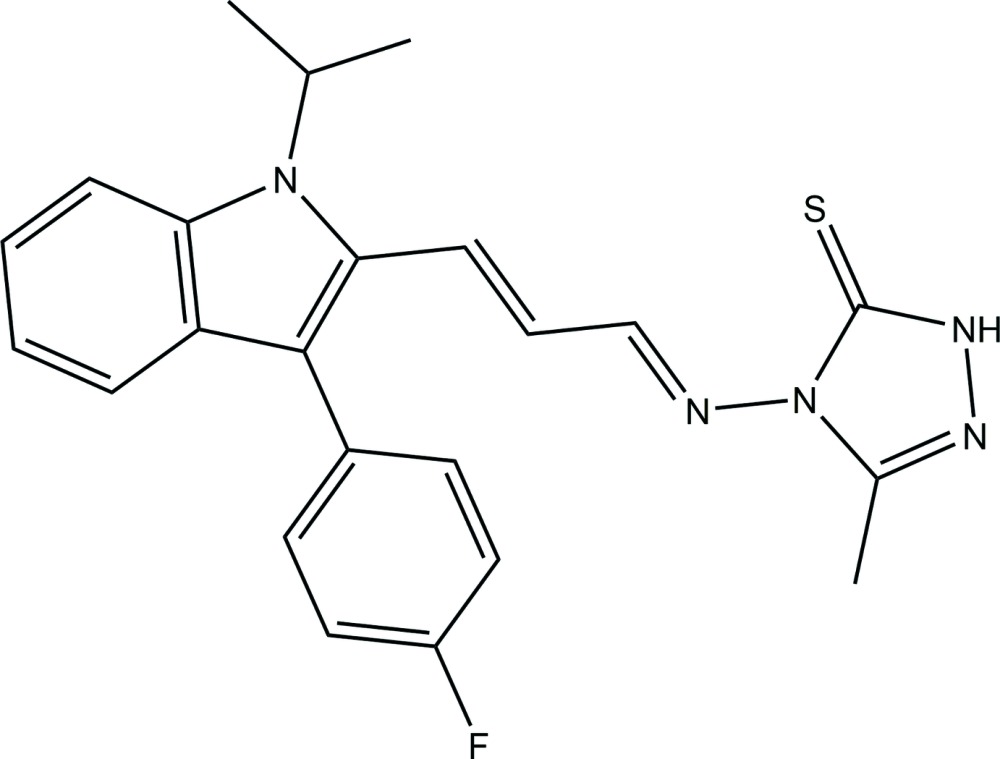


## Supra­molecular features   

**Figure 2:**
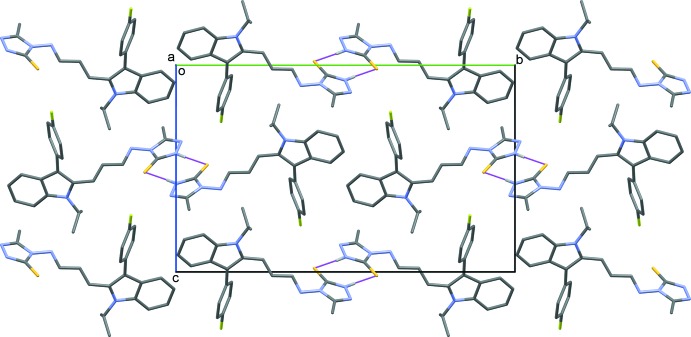
The crystal packing of the title compound viewed along the *a* axis. The N—H⋯S hydrogen bonds are shown as dashed lines (see Table 1 ▸). H atoms not involved in hydrogen bonding have been omitted for clarity.

In the crystal, mol­ecules are linked *via* pairs of N4—H4*B*⋯S1 hydrogen bonds, forming inversion dimers with an 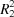
(8)ring motif (Table 1 ▸ and Fig. 2 ▸). The dimers are linked by C—H⋯π inter­actions (Table 1 ▸), forming chains along [1
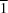
0]. The chains are linked by slipped parallel π–·π inter­actions involving inversion-related triazole rings [*Cg*2⋯*Cg*2^i^ = 3.4339 (13) Å; *Cg*2 is the centroid of the triazole ring (N3–N5/C12/C13); inter­planar distance = 3.3696 (8) Å, slippage = 0.662 Å; symmetry code: (i) −*x*, −*y* + 1, −*z* + 2], forming layers parallel to the *ab* plane.

## Database survey   

A search of the Cambridge Structural Database (CSD, Version 35.6, last update May 2015; Groom and Allen, 2014 ▸) revealed the presence of 60 structures containing the triazole-thione moiety but only four structures containing the fluvastatin [systematic name: (3*R*,5*S*,6*E*)-7-[3-(4-fluoro­phen­yl)-1-(propan-2-yl)-1*H*-indol-2-yl]-3,5-di­hydroxy­hept-6-enoic acid] nucleus. These include 5-[3-(4-fluoro­phen­yl)-1-isopropyl-1*H*-indol-2-yl]-1-(*X*)penta-2,4-diene-1-one (Kalalbandi *et al.*, 2015 ▸), where *X* = 4-nitro­phenyl (NUHNAH), 2-hy­droxy­phenyl (NUHNEL), 4-meth­oxy­phenyl (NUHNIP) and 4-chloro­phenyl (NUHNOV). In the four compounds, the 4-fluoro­phenyl ring of the fluvastatin nucleus is inclined to the indole ring by dihedral angles ranging from *ca* 46.66 to 68.59°, compared to 61.25 (8)° for the title compound.

## Synthesis and crystallization   

The title compound was synthesized following a reported procedure (Kulkarni *et al.*, 2011 ▸). A hot ethano­lic solution (60 ml) of 3-substituted-4-amino-5-mercapto-1,2,4-triazole (0.01 mol) and fluvastatin (0.01 mol) were refluxed for 4–5 h with addition of 4–5 drops of concentrated hydro­chloric acid. The precipitate obtained after evaporation of the solvent was filtered and washed with cold ethanol and recrystallized from hot ethanol. Crystals suitable for single-crystal diffraction analysis were obtained by slow evaporation of a solution in chloro­form (yield: 76%; m.p.: 469 K). ^1^H NMR (*d*_6_-DMSO): 10.6 (*s*, 1H, NH), 10.04 (*s*, 1H, CH=N), 7.1–7.6 (*m*, 8H, Ar–H), 6.47–6.56 (*d*, 2*H*, –CH=CH–), 2.38 (*s*, 1H, –CH_3_), 6.47–6.56 (*s*, 6H, isopropyl group). IR (KBr) cm^−1^: 3220, 3180 (N—H), 2753 (C—H), 1619 (C=N), 1500–1600 47 (C=C), 1102 (C=S). FAB MS: *m*/*z* 419. Elemental analysis: observed (calculated for C_23_H_22_FN_5_S) C, 65.94 (65.87); H, 5.19 (5.25); N, 16.66 (16.71) %.

## Refinement   

Crystal data, data collection and structure refinement details are summarized in Table 2 ▸. The N-bound H atom was located in a difference Fourier map and freely refined. The C-bound H atoms were positioned geometrically [C—H = 0.93–0.98 Å] and refined using a riding model with *U*_iso_(H) = 1.5*U*_eq_(C-meth­yl) and 1.2*U*_eq_(C) for other H atoms.

## Supplementary Material

Crystal structure: contains datablock(s) global, I. DOI: 10.1107/S2056989015020101/su5226sup1.cif

Structure factors: contains datablock(s) I. DOI: 10.1107/S2056989015020101/su5226Isup2.hkl

Supporting information file. DOI: 10.1107/S2056989015020101/su5226Isup3.cml

CCDC reference: 1433130

Additional supporting information:  crystallographic information; 3D view; checkCIF report

## Figures and Tables

**Table 1:** Hydrogen-bond geometry (, ) *Cg*1 is the centroid of the C18C23 ring.

*D*H*A*	*D*H	H*A*	*D* *A*	*D*H*A*
N4H4*B*S1^i^	0.91(2)	2.35(2)	3.257(2)	177.1(15)
C4H4*A* *Cg*1^ii^	0.93	2.93	3.724(2)	144

Symmetry codes: (i) 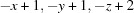; (ii) 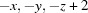.

**Table 2:** Experimental details

Crystal data	
Chemical formula	C_23_H_22_FN_5_S
*M*_r_	419.51
Crystal system, space group	Monoclinic, *P*2_1_/*c*
Temperature (K)	297
*a*, *b*, *c* ()	6.4388(8), 23.482(3), 14.572(3)
()	100.5009(19)
*V* (^3^)	2166.3(6)
*Z*	4
Radiation type	Mo *K*
(mm^1^)	0.18
Crystal size (mm)	0.40 0.27 0.09

Data collection	
Diffractometer	Bruker APEXII DUO CCD area-detector
Absorption correction	Multi-scan (*SADABS*; Bruker, 2009 ▸)
*T*_min_, *T*_max_	0.779, 0.932
No. of measured, independent and observed [*I* > 2(*I*)] reflections	24228, 5094, 3393
*R*_int_	0.041
(sin /)_max_ (^1^)	0.657

Refinement	
*R*[*F* ^2^ > 2(*F* ^2^)], *wR*(*F* ^2^), *S*	0.046, 0.127, 1.04
No. of reflections	5094
No. of parameters	278
H-atom treatment	H atoms treated by a mixture of independent and constrained refinement
_max_, _min_ (e ^3^)	0.24, 0.19

Computer programs: *APEX2* and *SAINT* (Bruker, 2009 ▸), *SHELXS97* (Sheldrick, 2008 ▸), *SHELXL2013* (Sheldrick, 2015 ▸), *Mercury* (Macrae *et al.*, 2008 ▸) and *PLATON* (Spek, 2009 ▸).

## supplementary crystallographic information

### Crystal data

**Table:**

C_23_H_22_FN_5_S	*F*(000) = 880
*M**_r_* = 419.51	*D*_x_ = 1.286 Mg m^−^^3^
Monoclinic, *P*2_1_/*c*	Mo *K*α radiation, λ = 0.71073 Å
*a* = 6.4388 (8) Å	Cell parameters from 5835 reflections
*b* = 23.482 (3) Å	θ = 2.8–27.5°
*c* = 14.572 (3) Å	µ = 0.18 mm^−^^1^
β = 100.5009 (19)°	*T* = 297 K
*V* = 2166.3 (6) Å^3^	Block, yellow
*Z* = 4	0.40 × 0.27 × 0.09 mm

### Data collection

**Table:**

Bruker APEXII DUO CCD area-detector diffractometer	5094 independent reflections
Radiation source: fine-focus sealed tube	3393 reflections with *I* > 2σ(*I*)
Graphite monochromator	*R*_int_ = 0.041
φ and ω scans	θ_max_ = 27.8°, θ_min_ = 1.7°
Absorption correction: multi-scan (*SADABS*; Bruker, 2009)	*h* = −8→8
*T*_min_ = 0.779, *T*_max_ = 0.932	*k* = −30→30
24228 measured reflections	*l* = −19→18

### Refinement

**Table:**

Refinement on *F*^2^	Primary atom site location: structure-invariant direct methods
Least-squares matrix: full	Secondary atom site location: difference Fourier map
*R*[*F*^2^ > 2σ(*F*^2^)] = 0.046	Hydrogen site location: mixed
*wR*(*F*^2^) = 0.127	H atoms treated by a mixture of independent and constrained refinement
*S* = 1.04	*w* = 1/[σ^2^(*F*_o_^2^) + (0.0559*P*)^2^ + 0.3794*P*] where *P* = (*F*_o_^2^ + 2*F*_c_^2^)/3
5094 reflections	(Δ/σ)_max_ = 0.001
278 parameters	Δρ_max_ = 0.24 e Å^−^^3^
0 restraints	Δρ_min_ = −0.19 e Å^−^^3^

### Special details

**Table:**

Geometry. All e.s.d.'s (except the e.s.d. in the dihedral angle between two l.s. planes) are estimated using the full covariance matrix. The cell e.s.d.'s are taken into account individually in the estimation of e.s.d.'s in distances, angles and torsion angles; correlations between e.s.d.'s in cell parameters are only used when they are defined by crystal symmetry. An approximate (isotropic) treatment of cell e.s.d.'s is used for estimating e.s.d.'s involving l.s. planes.

### Fractional atomic coordinates and isotropic or equivalent isotropic displacement parameters (Å^2^)

**Table:**

	*x*	*y*	*z*	*U*_iso_*/*U*_eq_	
S1	0.39854 (8)	0.40778 (2)	0.98203 (4)	0.06099 (17)	
F1	0.5717 (2)	0.13276 (6)	1.27264 (9)	0.0912 (4)	
N1	−0.3776 (2)	0.17803 (6)	0.86118 (10)	0.0523 (4)	
N2	0.0328 (2)	0.36814 (6)	1.09831 (11)	0.0512 (4)	
N3	0.0953 (2)	0.42497 (6)	1.09021 (10)	0.0464 (3)	
N4	0.2694 (3)	0.49906 (7)	1.07257 (11)	0.0540 (4)	
H4B	0.358 (3)	0.5255 (9)	1.0558 (15)	0.073 (7)*	
N5	0.1315 (3)	0.51498 (7)	1.13047 (11)	0.0564 (4)	
C1	−0.4000 (3)	0.12025 (8)	0.87117 (12)	0.0497 (4)	
C2	−0.5378 (3)	0.08101 (9)	0.81951 (14)	0.0598 (5)	
H2A	−0.6413	0.0929	0.7702	0.072*	
C3	−0.5163 (3)	0.02485 (9)	0.84345 (15)	0.0655 (6)	
H3A	−0.6061	−0.0017	0.8093	0.079*	
C4	−0.3635 (3)	0.00615 (9)	0.91768 (15)	0.0636 (5)	
H4A	−0.3509	−0.0325	0.9313	0.076*	
C5	−0.2315 (3)	0.04425 (8)	0.97074 (14)	0.0547 (5)	
H5A	−0.1318	0.0318	1.0211	0.066*	
C6	−0.2489 (3)	0.10201 (7)	0.94809 (12)	0.0457 (4)	
C7	−0.1334 (3)	0.15099 (7)	0.98611 (11)	0.0446 (4)	
C8	−0.2146 (3)	0.19687 (8)	0.93150 (12)	0.0474 (4)	
C9	−0.1544 (3)	0.25633 (8)	0.93989 (13)	0.0529 (4)	
H9A	−0.1696	0.2772	0.8848	0.063*	
C10	−0.0801 (3)	0.28383 (8)	1.01813 (13)	0.0503 (4)	
H10A	−0.0641	0.2640	1.0743	0.060*	
C11	−0.0232 (3)	0.34278 (8)	1.02010 (13)	0.0498 (4)	
H11A	−0.0270	0.3626	0.9646	0.060*	
C12	0.2548 (3)	0.44453 (8)	1.04702 (12)	0.0478 (4)	
C13	0.0300 (3)	0.46845 (8)	1.14103 (13)	0.0507 (4)	
C14	−0.1374 (3)	0.46154 (10)	1.19744 (16)	0.0702 (6)	
H14A	−0.2661	0.4498	1.1578	0.105*	
H14B	−0.1598	0.4971	1.2265	0.105*	
H14C	−0.0951	0.4332	1.2446	0.105*	
C15	−0.4963 (3)	0.21375 (9)	0.78577 (13)	0.0592 (5)	
H15A	−0.4358	0.2520	0.7967	0.071*	
C16	−0.7234 (4)	0.22020 (12)	0.79263 (17)	0.0881 (8)	
H16A	−0.7933	0.2432	0.7418	0.132*	
H16B	−0.7342	0.2382	0.8508	0.132*	
H16C	−0.7888	0.1833	0.7897	0.132*	
C17	−0.4518 (4)	0.19635 (11)	0.69253 (15)	0.0850 (7)	
H17A	−0.5220	0.2219	0.6456	0.127*	
H17B	−0.5024	0.1583	0.6785	0.127*	
H17C	−0.3023	0.1976	0.6937	0.127*	
C18	0.0509 (3)	0.14878 (7)	1.06333 (12)	0.0446 (4)	
C19	0.0324 (3)	0.12700 (8)	1.15036 (13)	0.0536 (5)	
H19A	−0.0996	0.1158	1.1611	0.064*	
C20	0.2057 (3)	0.12172 (9)	1.22087 (14)	0.0604 (5)	
H20A	0.1915	0.1075	1.2789	0.072*	
C21	0.3980 (3)	0.13781 (9)	1.20369 (14)	0.0602 (5)	
C22	0.4252 (3)	0.15878 (9)	1.11945 (15)	0.0624 (5)	
H22A	0.5585	0.1693	1.1094	0.075*	
C23	0.2508 (3)	0.16406 (8)	1.04971 (13)	0.0548 (5)	
H23A	0.2676	0.1783	0.9919	0.066*	

### Atomic displacement parameters (Å^2^)

**Table:**

	*U*^11^	*U*^22^	*U*^33^	*U*^12^	*U*^13^	*U*^23^
S1	0.0679 (3)	0.0568 (3)	0.0619 (3)	−0.0073 (2)	0.0215 (3)	−0.0065 (2)
F1	0.0731 (8)	0.1057 (11)	0.0794 (9)	0.0101 (7)	−0.0271 (7)	0.0028 (8)
N1	0.0596 (9)	0.0492 (9)	0.0423 (8)	0.0011 (7)	−0.0065 (7)	0.0004 (7)
N2	0.0588 (9)	0.0437 (8)	0.0510 (9)	−0.0046 (7)	0.0099 (7)	−0.0012 (7)
N3	0.0505 (8)	0.0427 (8)	0.0439 (8)	−0.0035 (6)	0.0032 (7)	−0.0005 (6)
N4	0.0575 (9)	0.0487 (9)	0.0558 (10)	−0.0077 (8)	0.0106 (8)	−0.0006 (7)
N5	0.0577 (9)	0.0509 (9)	0.0594 (10)	−0.0023 (8)	0.0073 (8)	−0.0055 (8)
C1	0.0537 (10)	0.0520 (11)	0.0423 (10)	−0.0005 (8)	0.0059 (8)	−0.0043 (8)
C2	0.0636 (12)	0.0635 (13)	0.0485 (11)	−0.0081 (10)	0.0005 (9)	−0.0069 (9)
C3	0.0713 (13)	0.0632 (13)	0.0612 (13)	−0.0189 (11)	0.0100 (11)	−0.0146 (10)
C4	0.0787 (14)	0.0463 (11)	0.0681 (13)	−0.0077 (10)	0.0197 (12)	−0.0062 (10)
C5	0.0589 (11)	0.0493 (11)	0.0564 (11)	0.0029 (9)	0.0121 (9)	0.0011 (9)
C6	0.0490 (10)	0.0459 (10)	0.0424 (9)	0.0021 (8)	0.0091 (8)	−0.0026 (8)
C7	0.0495 (10)	0.0432 (9)	0.0402 (9)	0.0031 (8)	0.0061 (8)	−0.0008 (7)
C8	0.0523 (10)	0.0471 (10)	0.0407 (9)	0.0010 (8)	0.0023 (8)	−0.0023 (8)
C9	0.0603 (11)	0.0470 (10)	0.0474 (10)	0.0030 (8)	−0.0005 (9)	0.0053 (8)
C10	0.0557 (10)	0.0455 (10)	0.0495 (10)	0.0001 (8)	0.0091 (8)	0.0010 (8)
C11	0.0499 (10)	0.0483 (10)	0.0494 (11)	−0.0007 (8)	0.0040 (8)	0.0022 (8)
C12	0.0503 (10)	0.0486 (10)	0.0416 (9)	−0.0048 (8)	0.0007 (8)	0.0028 (8)
C13	0.0515 (10)	0.0502 (11)	0.0481 (10)	0.0006 (8)	0.0029 (8)	−0.0056 (8)
C14	0.0722 (13)	0.0654 (13)	0.0783 (15)	−0.0021 (11)	0.0277 (12)	−0.0120 (11)
C15	0.0663 (12)	0.0599 (12)	0.0457 (11)	0.0063 (10)	−0.0046 (9)	0.0045 (9)
C16	0.0896 (17)	0.0990 (19)	0.0737 (15)	0.0358 (15)	0.0092 (13)	0.0178 (14)
C17	0.1087 (19)	0.0920 (18)	0.0548 (13)	0.0203 (15)	0.0165 (13)	0.0172 (12)
C18	0.0495 (10)	0.0391 (9)	0.0436 (9)	0.0042 (7)	0.0041 (8)	−0.0019 (7)
C19	0.0544 (10)	0.0573 (11)	0.0481 (10)	0.0006 (9)	0.0064 (8)	0.0038 (9)
C20	0.0727 (13)	0.0614 (12)	0.0437 (10)	0.0064 (10)	0.0016 (9)	0.0065 (9)
C21	0.0584 (12)	0.0567 (12)	0.0572 (12)	0.0080 (9)	−0.0111 (9)	−0.0031 (10)
C22	0.0464 (10)	0.0647 (13)	0.0736 (14)	0.0022 (9)	0.0042 (10)	−0.0006 (11)
C23	0.0555 (11)	0.0580 (11)	0.0508 (11)	0.0036 (9)	0.0094 (9)	0.0054 (9)

### Geometric parameters (Å, º)

**Table:**

S1—C12	1.6785 (19)	C9—H9A	0.9300
F1—C21	1.365 (2)	C10—C11	1.431 (3)
N1—C1	1.375 (2)	C10—H10A	0.9300
N1—C8	1.398 (2)	C11—H11A	0.9300
N1—C15	1.480 (2)	C13—C14	1.479 (3)
N2—C11	1.278 (2)	C14—H14A	0.9600
N2—N3	1.405 (2)	C14—H14B	0.9600
N3—C13	1.372 (2)	C14—H14C	0.9600
N3—C12	1.377 (2)	C15—C16	1.491 (3)
N4—C12	1.332 (2)	C15—C17	1.496 (3)
N4—N5	1.383 (2)	C15—H15A	0.9800
N4—H4B	0.91 (2)	C16—H16A	0.9600
N5—C13	1.297 (2)	C16—H16B	0.9600
C1—C2	1.400 (3)	C16—H16C	0.9600
C1—C6	1.410 (2)	C17—H17A	0.9600
C2—C3	1.365 (3)	C17—H17B	0.9600
C2—H2A	0.9300	C17—H17C	0.9600
C3—C4	1.394 (3)	C18—C23	1.385 (2)
C3—H3A	0.9300	C18—C19	1.393 (2)
C4—C5	1.371 (3)	C19—C20	1.377 (3)
C4—H4A	0.9300	C19—H19A	0.9300
C5—C6	1.396 (3)	C20—C21	1.361 (3)
C5—H5A	0.9300	C20—H20A	0.9300
C6—C7	1.426 (2)	C21—C22	1.363 (3)
C7—C8	1.384 (2)	C22—C23	1.376 (3)
C7—C18	1.480 (2)	C22—H22A	0.9300
C8—C9	1.448 (3)	C23—H23A	0.9300
C9—C10	1.322 (2)		
			
C1—N1—C8	108.31 (14)	N3—C12—S1	128.32 (14)
C1—N1—C15	126.00 (15)	N5—C13—N3	110.63 (16)
C8—N1—C15	125.60 (16)	N5—C13—C14	126.34 (17)
C11—N2—N3	113.96 (15)	N3—C13—C14	123.02 (17)
C13—N3—C12	109.04 (15)	C13—C14—H14A	109.5
C13—N3—N2	122.74 (14)	C13—C14—H14B	109.5
C12—N3—N2	127.16 (15)	H14A—C14—H14B	109.5
C12—N4—N5	114.22 (16)	C13—C14—H14C	109.5
C12—N4—H4B	126.7 (14)	H14A—C14—H14C	109.5
N5—N4—H4B	119.1 (14)	H14B—C14—H14C	109.5
C13—N5—N4	103.77 (15)	N1—C15—C16	112.83 (18)
N1—C1—C2	131.40 (17)	N1—C15—C17	111.16 (17)
N1—C1—C6	108.25 (15)	C16—C15—C17	116.2 (2)
C2—C1—C6	120.35 (18)	N1—C15—H15A	105.2
C3—C2—C1	118.28 (19)	C16—C15—H15A	105.2
C3—C2—H2A	120.9	C17—C15—H15A	105.2
C1—C2—H2A	120.9	C15—C16—H16A	109.5
C2—C3—C4	121.82 (19)	C15—C16—H16B	109.5
C2—C3—H3A	119.1	H16A—C16—H16B	109.5
C4—C3—H3A	119.1	C15—C16—H16C	109.5
C5—C4—C3	120.6 (2)	H16A—C16—H16C	109.5
C5—C4—H4A	119.7	H16B—C16—H16C	109.5
C3—C4—H4A	119.7	C15—C17—H17A	109.5
C4—C5—C6	119.07 (19)	C15—C17—H17B	109.5
C4—C5—H5A	120.5	H17A—C17—H17B	109.5
C6—C5—H5A	120.5	C15—C17—H17C	109.5
C5—C6—C1	119.82 (17)	H17A—C17—H17C	109.5
C5—C6—C7	132.71 (17)	H17B—C17—H17C	109.5
C1—C6—C7	107.42 (15)	C23—C18—C19	117.50 (17)
C8—C7—C6	106.79 (15)	C23—C18—C7	121.29 (16)
C8—C7—C18	129.11 (16)	C19—C18—C7	121.02 (16)
C6—C7—C18	123.81 (15)	C20—C19—C18	121.35 (18)
C7—C8—N1	109.23 (15)	C20—C19—H19A	119.3
C7—C8—C9	129.40 (16)	C18—C19—H19A	119.3
N1—C8—C9	121.36 (16)	C21—C20—C19	118.57 (18)
C10—C9—C8	126.37 (17)	C21—C20—H20A	120.7
C10—C9—H9A	116.8	C19—C20—H20A	120.7
C8—C9—H9A	116.8	C20—C21—C22	122.43 (18)
C9—C10—C11	122.75 (17)	C20—C21—F1	119.42 (19)
C9—C10—H10A	118.6	C22—C21—F1	118.15 (19)
C11—C10—H10A	118.6	C21—C22—C23	118.45 (19)
N2—C11—C10	119.87 (17)	C21—C22—H22A	120.8
N2—C11—H11A	120.1	C23—C22—H22A	120.8
C10—C11—H11A	120.1	C22—C23—C18	121.70 (18)
N4—C12—N3	102.30 (15)	C22—C23—H23A	119.2
N4—C12—S1	129.35 (14)	C18—C23—H23A	119.2
			
C11—N2—N3—C13	135.05 (17)	N3—N2—C11—C10	176.73 (15)
C11—N2—N3—C12	−58.1 (2)	C9—C10—C11—N2	174.77 (18)
C12—N4—N5—C13	0.3 (2)	N5—N4—C12—N3	1.0 (2)
C8—N1—C1—C2	−179.70 (19)	N5—N4—C12—S1	−177.04 (13)
C15—N1—C1—C2	−3.0 (3)	C13—N3—C12—N4	−1.95 (18)
C8—N1—C1—C6	−0.5 (2)	N2—N3—C12—N4	−170.26 (15)
C15—N1—C1—C6	176.20 (16)	C13—N3—C12—S1	176.15 (14)
N1—C1—C2—C3	176.7 (2)	N2—N3—C12—S1	7.8 (2)
C6—C1—C2—C3	−2.4 (3)	N4—N5—C13—N3	−1.6 (2)
C1—C2—C3—C4	0.6 (3)	N4—N5—C13—C14	179.73 (18)
C2—C3—C4—C5	1.4 (3)	C12—N3—C13—N5	2.3 (2)
C3—C4—C5—C6	−1.5 (3)	N2—N3—C13—N5	171.27 (15)
C4—C5—C6—C1	−0.4 (3)	C12—N3—C13—C14	−178.93 (17)
C4—C5—C6—C7	−177.33 (19)	N2—N3—C13—C14	−10.0 (3)
N1—C1—C6—C5	−176.99 (16)	C1—N1—C15—C16	69.7 (3)
C2—C1—C6—C5	2.3 (3)	C8—N1—C15—C16	−114.2 (2)
N1—C1—C6—C7	0.7 (2)	C1—N1—C15—C17	−62.8 (3)
C2—C1—C6—C7	−179.99 (17)	C8—N1—C15—C17	113.3 (2)
C5—C6—C7—C8	176.61 (19)	C8—C7—C18—C23	−58.3 (3)
C1—C6—C7—C8	−0.63 (19)	C6—C7—C18—C23	114.7 (2)
C5—C6—C7—C18	2.3 (3)	C8—C7—C18—C19	126.8 (2)
C1—C6—C7—C18	−174.99 (15)	C6—C7—C18—C19	−60.2 (2)
C6—C7—C8—N1	0.35 (19)	C23—C18—C19—C20	1.1 (3)
C18—C7—C8—N1	174.31 (16)	C7—C18—C19—C20	176.15 (17)
C6—C7—C8—C9	179.80 (18)	C18—C19—C20—C21	−0.6 (3)
C18—C7—C8—C9	−6.2 (3)	C19—C20—C21—C22	−0.1 (3)
C1—N1—C8—C7	0.1 (2)	C19—C20—C21—F1	−179.84 (17)
C15—N1—C8—C7	−176.62 (17)	C20—C21—C22—C23	0.4 (3)
C1—N1—C8—C9	−179.43 (17)	F1—C21—C22—C23	−179.90 (18)
C15—N1—C8—C9	3.9 (3)	C21—C22—C23—C18	0.1 (3)
C7—C8—C9—C10	−31.3 (3)	C19—C18—C23—C22	−0.8 (3)
N1—C8—C9—C10	148.07 (19)	C7—C18—C23—C22	−175.87 (17)
C8—C9—C10—C11	179.64 (17)		

### Hydrogen-bond geometry (Å, º)

Cg1 is the centroid of the C18–C23 ring.

**Table:**

*D*—H···*A*	*D*—H	H···*A*	*D*···*A*	*D*—H···*A*
N4—H4*B*···S1^i^	0.91 (2)	2.35 (2)	3.257 (2)	177.1 (15)
C4—H4*A*···*Cg*1^ii^	0.93	2.93	3.724 (2)	144

Symmetry codes: (i) −*x*+1, −*y*+1, −*z*+2; (ii) −*x*, −*y*, −*z*+2.
